# Fellow-Workers and Their Doings

**Published:** 1914-05-16

**Authors:** 


### ROUND THE WORLD.

### Fellow-Workers and their Doings.

[The Editor Invites Early Information and Contributions Marked "Fellow-Workers."]

Dr. Arthur Cyril Hudson is to be congratulated on his recognition as a teacher in ophthalmology by the senate of London University. His official tutorial work will, of course, be carried out at the Royal Ophthalmic Hospital (Moorfields), where he is an assistant surgeon. Dr. Hudson, who is an M.D., B.S. Cantab. and F.R.C.S. Eng., is ophthalmological registrar at St. Thomas's Hospital, where he had a brilliant career as a student, entering with a University scholarship in 1899.

Dr. Basil T. Parsons-Smith, the fortunate recipient of the Hunterian Society's medal, has for some time been interested in circulatory problems and embodied the results of numerous observations on the phenomena of intermittent pulse in his prize essay.

Dr. J. A. Ormerod, M.D., F.R.C.P., in his recent Lumleian lectures at the Royal College of Physicians, has summarised the symptoms and modern theories of hysteria in a way that cannot fail to be of extreme use to the general physician, and many practitioners will undoubtedly look forward to reading his remarks in book form. Dr. Ormerod has had a distinguished career, being for many years associated with the active work at St. Bartholomew's Hospital and the National Hospital for the Paralysed and Epileptic in Queen Square, to both of which institutions he is now consulting physician; he is registrar to the Royal College of Physicians, for which body he delivered the Harveian Oration in 1908; and formerly was on the staff of the City of London Hospital for Diseases of the Chest.

Sir Robert Michael Simon, who has just retired from the post of senior physician to the Birmingham General Hospital, has served on the active staff of that institution for over twenty years. Qualifying M.R.C.S. from Cambridge and Guy's in 1875, this well-known Midland consultant subsequently obtained the higher professional distinctions of M.D. Cantab. and M.R.C.P. Lond., being elected to the fellowship of the Royal College of Physicians in 1895. Formerly physician to the Manchester Southern Hospital for Women and Children, Dr. Simon subsequently settled in Birmingham, where he was given numerous responsible appointments, including that of professor of therapeutics in Birmingham University. Sir Robert Simon, who finds his recreation in golf and chess, has written on industrial diseases, dermatology, and general medicine; he received the honour of knighthood four years ago.

Mr. Robert Foster Moore, M.B., F.R.C.S., who has just been elected to the staff of the Royal London Ophthalmic Hospital (Moorfields), is one of the assistant surgeons at the Central London Ophthalmic Hospital, and has for some years been working in the eye department at "Bart.'s," where he passed his student days. Mr. Moore's academic distinctions include scholarships at Cambridge, St. Bartholomew's, and the Lang Scholarship at "Moorfields."

Professor J. M. Beattie's recent work on the electrical treatment of milk is of the greatest interest to all institutional authorities, suggesting as it does the principles on which sterilisation may be universally carried out in the future. Professor Beattie is, of course, bacteriologist to the city of Liverpool, as well as professor of bacteriology in the University there. Having received his medical training at Otago and Edinburgh, this prominent investigator gained the gold medal at the M.D. Examination of the latter University in 1901.

The new honorary radiologist to the Croydon General Hospital, Dr. Frederick Harwood-Hardman, M.D. Edin., is an old London Hospital man. Formerly on the medical staff of the Cunard Company, Dr. Harwood-Hardman interested himself in radiography, and has lately been working as chief assistant in the *x*-ray department of the West London Hospital, and as clinical assistant in the electro-therapeutic department at that institution as well as at St. John's Hospital for Skin Diseases.

Dr. Robert Armstrong-Jones, whose address on the care of the insane at the recent Nursing Conference was very much to the point, is to-day one of our first authorities on mental disease, and as superintendent of the huge asylum at Claybury has considerably advanced our methods of dealing with persons of unsound mind. Formerly lecturer on mental diseases at Westminster Hospital, Dr. Armstrong-Jones, who is an M.D. and F.R.C.P. Lond., now occupies a similar position at "Bart.'s," where he received his early medical education.

Dr. Charles Edgar Lea is to be congratulated on his appointment as honorary physician to the Ancoats Hospital, Manchester. Receiving his medical training at the Victoria University, Manchester, and at King's College, London, Dr. Lea, who is honorary assistant physician to the Salford Royal Hospital and medical registrar at the Manchester Royal Infirmary, became an M.D. Vict. Manch. in 1909 and M.R.C.P. Lond. in 1912. He has written extensively on heart disease, in connection with which he has made many original observations, having given special reference to abnormalities of the cardiac rhythm.

Mr. Harry Platt, M.S. Lond., F.R.C.S. Eng., who has been elected honorary surgeon to the same institution, studied medicine both in Manchester and at the London Hospital, having been awarded the University gold medal, with distinctions in medicine and surgery, at the M.B., B.S. Examination of London University in 1909. Since qualification, Mr. Platt has had plenty of clinical experience, both as house surgeon to the Manchester Royal Infirmary and the Royal National Orthopædic Hospital, as well as in the capacity of clinical assistant at St. Peter's Hospital for Stone and the Queen's Hospital for Children.

Dr. S. E. Dore, M.D., M.R.C.P., whose appointment as consulting physician for skin diseases at the Evelina Hospital for Sick Children is announced, has been largely instrumental in developing radium treatment in this country, as well as the *x*-ray treatment of ringworm and other maladies of the skin. For many years his work has been closely associated with that of Sir Malcolm Morris, who is generally recognised as one of the foremost living dermatologists. Dr. Dore, who is an old student of Cambridge and St. Mary's, occupies the posts of assistant physician for skin diseases at Westminster Hospital, where he is lecturer on dermatology, and physician to the skin department at the Hampstead General Hospital.

